# Exhaled Nitric Oxide and Olfactory Dysfunction in Patients with Asthma: Association with Chronic Rhinosinusitis

**DOI:** 10.3390/medicina59101776

**Published:** 2023-10-05

**Authors:** Takashi Oda, Hiroshi Iwamoto, Sachio Takeno, Tomohiro Kawasumi, Kota Takemoto, Manabu Nishida, Nobuyuki Chikuie, Yuichiro Horibe, Kakuhiro Yamaguchi, Shinjiro Sakamoto, Naoko Higaki, Takayuki Taruya, Yasushi Horimasu, Takeshi Masuda, Takao Hamamoto, Taku Nakashima, Takashi Ishino, Tsutomu Ueda, Kazunori Fujitaka, Hironobu Hamada, Noboru Hattori

**Affiliations:** 1 Department of Otorhinolaryngology, Head & Neck Surgery, Graduate School of Biomedical & Health Sciences, Hiroshima University, 1-2-3 Kasumi, Minami-ku, Hiroshima 734-8551, Japan; odataka@hiroshima-u.ac.jp (T.O.); kwtm2022@hiroshima-u.ac.jp (T.K.); kota61@hiroshima-u.ac.jp (K.T.); nm1027@hiroshima-u.ac.jp (M.N.); housejak@hiroshima-u.ac.jp (N.C.); horibey@hiroshima-u.ac.jp (Y.H.); ttaruya@hiroshima-u.ac.jp (T.T.); takao0320@hiroshima-u.ac.jp (T.H.); tishino@hiroshima-u.ac.jp (T.I.); uedatsu@hiroshima-u.ac.jp (T.U.); 2 Department of Molecular and Internal Medicine, Graduate School of Biomedical and Health Sciences, Hiroshima University, 1-2-3 Kasumi, Minami-ku, Hiroshima 734-8551, Japan; iwamotohiroshig@gmail.com (H.I.); yamaguchikakuhiro@gmail.com (K.Y.); s-sakamoto@hiroshima-u.ac.jp (S.S.); na.higaki@gmail.com (N.H.); yasushi17@hiroshima-u.ac.jp (Y.H.); ta-masuda@hiroshima-u.ac.jp (T.M.); tnaka@hiroshima-u.ac.jp (T.N.); fujikazu@hiroshima-u.ac.jp (K.F.); nhattori@hiroshima-u.ac.jp (N.H.); 3 Department of Physical Analysis and Therapeutic Sciences, Graduate School of Biomedical and Health Sciences, Hiroshima University, Hiroshima 734-8551, Japan; hirohamada@hiroshima-u.ac.jp

**Keywords:** asthma, sinusitis, olfactory dysfunction, fractional exhaled nitric oxide, eosinophils

## Abstract

*Objectives*: Olfactory dysfunction is a clinical sign that is important to detect with coexistent upper airway comorbidities in patients with asthma. This study aimed to investigate the etiology of olfactory dysfunction in patients with asthma and the relationship between fractional exhaled nitric oxide (FeNO) levels. *Materials and Methods*: This study included 47 asthma patients who were evaluated for olfactory dysfunction at Hiroshima University Hospital between 2012 and 2020. The etiologies of olfactory dysfunction were evaluated, and they were classified according to the FeNO levels of patients with asthma. *Results*: Olfactory dysfunction was observed in 30 patients with asthma, with chronic rhinosinusitis (77%) being the most prevalent etiology. Eosinophilic chronic rhinosinusitis (ECRS) was the most prevalent etiology of olfactory dysfunction in asthma patients with high FeNO levels (≥25 ppb), while non-eosinophilic chronic rhinosinusitis (NCRS) was the most prevalent etiology in asthma patients with low FeNO levels (<25 ppb). Additionally, the prevalence of ECRS was significantly higher in asthma patients with olfactory dysfunction and high FeNO levels (74%) than in those with either high FeNO levels or olfactory dysfunction and those with low FeNO levels and no olfactory dysfunction (12% and 9%, respectively). *Conclusions*: We found that ECRS was the predominant cause of olfactory dysfunction in patients with high FeNO levels, while NCRS was more common in those with low FeNO levels. The present study showed that both ECRS and NCRS are common etiologies of olfactory dysfunction in patients with asthma. Additionally, this study supports the link between upper and lower airway inflammation in patients with asthma complicated with olfactory dysfunction.

## 1. Introduction

Olfaction is one of the most important sensations in humans to maintain a high quality of daily life, and it is also essential to avoid danger signals around residual environments. Olfactory dysfunction is a clinical sign that is important to detect with coexistent upper airway comorbidities in patients with asthma. The etiology of olfactory dysfunction is diverse, and over 200 causes of olfactory dysfunction have been reported; frequent causes of olfactory dysfunction in the general population have been reported to be idiopathic, rhinitis, sinus infection, upper respiratory tract infection, and head trauma [1,2]. Because these different pathophysiological mechanisms require different examination and treatment modalities, appropriate and individual diagnoses are necessary. In respect to diagnostic tools of human airway diseases, it would be helpful for clinicians to know the prevalence of the underlying diseases that cause olfactory dysfunction in patients with asthma. However, to date, the prevalence of olfactory dysfunction etiologies in patients with asthma has not been reported. 

Chronic rhinosinusitis (CRS) is a heterogeneous disease caused by various inflammatory mechanisms with a considerable social burden [3]. It is defined as symptomatic inflammation of the sinonasal mucosa with evidence of inflammation on endoscopic exams and/or imaging lasting more than 12 weeks. Patients with CRS complain of purulent nasal discharge, nasal obstruction, headaches, cheek pain, and toothaches. In Japan, this disorder was once called empyema rather than CRS. Some CRS patients develop polyps in the nasal cavity, while others do not [4]. Eosinophilic CRS (ECRS) is a subgroup of refractory CRS characterized by the presence of nasal polyps with eosinophil infiltration in the ethmoidal sinus, frequent olfactory disturbance, and resistance to treatment [5,6]. Eosinophilic CRS is classified as a CRS phenotype with dominant type 2 inflammation. Patients with ECRS are characterized by massive nasal polyps in bilateral nasal cavities with ethmoid-predominant sinus opacification and tissue eosinophil infiltration. The patients generally show a poor response to medical and surgical treatments in comparison with those with non-eosinophilic CRS (NCRS). Persistent symptoms of severe ECRS include nasal blockage, nasal discharge, and olfactory dysfunction, with patients frequently reporting an altered sense of smell and taste. Previous studies showed that 34.7% of patients with ECRS had comorbid asthma, and the combination of asthma and ECRS is a risk factor for the severity and refractoriness of each condition [3,7,8,9]. Additionally, NCRS was observed in patients with asthma, and the coexistence of upper airway diseases could impact the treatment strategies for asthma [10]. 

Interestingly, we and others have shown that fractional exhaled nitric oxide (FeNO) levels are elevated in patients with ECRS, irrespective of comorbid asthma [11,12,13]. Nitric oxide (NO), a paramagnetic molecule with an odd number of electrons, is a radical with extreme reactivity that is responsible for many of its biological effects. Cellular signals transmitted by NO are important in the regulation of a variety of physiological and pathological functions, including those for the nervous, vascular, and respiratory systems. In human airways, NO is well-known to have both physiological and inflammatory roles through the production of various cell types including structural and migrating inflammatory cells. Nitric oxide stimulates cell proliferation, migration, differentiation, and immune responses. Further, the measurement of FeNO levels is a well-established biomarker for type 2 inflammation in bronchial asthma (BA), and the FeNO levels decrease in response to medical interventions such as treatment with inhaled corticosteroids (ICS) or anti-IL-4/-IL-13R antibodies. We hypothesized that the types and frequencies of etiologies of olfactory dysfunction, such as ECRS and NCRS, would differ according to the FeNO values among patients with asthma.

This study, therefore, aimed to investigate the etiology of olfactory dysfunction in patients with asthma and to assess the relationship between FeNO levels and the etiology of olfactory dysfunction. For this purpose, a group of asthma patients was evaluated for olfactory dysfunction and was classified by FeNO levels. We found that ECRS comorbidity was the most prevalent etiology of olfactory dysfunction in the asthma patients with higher FeNO levels. The results further substantiated the clinical significance of the prevalence of both ECRS and NCRS as common etiologies of olfactory dysfunction in asthma patients and implied intimate links between upper and lower eosinophilic airway inflammation.

## 2. Material and Methods

### 2.1. Patient Enrollment

This study included 47 adult asthma patients who had been evaluated for olfactory dysfunction at Hiroshima University Hospital between 2012 and 2020. All the patients were Japanese. Clinical records and laboratory data were collected from the patients’ medical records. All procedures contributing to this work complied with the ethical standards of the Helsinki Declaration of 1975, revised in 2013. The Medical Ethics Committee of Hiroshima University approved this study (E-2033) and waived the requirement for obtaining signed informed consent as this was a retrospective observational study.

### 2.2. Study Assessment

Patient backgrounds and characteristics, including age, sex, body mass index (BMI), asthma control test (ACT) score, asthma control questionnaire (ACQ) score, asthma medication use, pulmonary function test results, FeNO levels, and blood eosinophil counts, were collected. Olfactory dysfunction was evaluated using a T&T olfactometer, the SNOT-22, and medical interviews (Table 1). 

T&T olfactometry (Daiichi Yakuhin Sangyo, Tokyo, Japan) has been commonly used in Japan for evaluating patients with olfactory dysfunction. The device utilizes 5 distinct odors, i.e., b-phenylethyl alcohol, methyl cyclopentenolone, isovaleric acid, g-undecalactone, and skatole, with 7–8 graduated concentration levels. Odor detection and cognitive thresholds are recorded for each element, with a normal cognitive threshold being less than 1.0. The severity of olfactory dysfunction is classified by the mean T&T cognitive threshold: a value of 1.0 or less indicates no dysfunction, 1.1–2.5 is mild, 2.6–4.0 is moderate, 4.1–5.5 is severe, and 5.6 or more is labeled as anosmia [14]. The judgment of olfactory disorder was also rendered based on the question item “Loss of smell or taste” on the SNOT-22.

During this study period, because the same patient may have had more than one form of olfactory dysfunction (anosmia, hyposmia, or parosmia) at different stages of the disease, we considered any form of smell disorder to be olfactory dysfunction [15]. Atopic status was defined as a positive specific IgE antibody response to common aeroallergens. Allergic rhinitis was diagnosed by physicians based on its clinical features (frequent rhinorrhea, nasal obstruction, and mucosal swelling). Pulmonary function was measured using spirometry, and the percentage of the predicted values was calculated using the Japanese reference values [16]. FeNO levels were analyzed using a NIOX VERO^®^ (Aerocrine AB, Solna, Sweden) following the recommendations of the European Respiratory Society/American Thoracic Society [17]. The nasal polyp score was separately assessed with an endoscope for each nostril. The highest score per side was graded. Each nostril was scored from 0–4 (0 = no polyps, 1 = small polyps confined to the middle meatus, 2 = blocked middle meatus, 3 = polyps extending beyond the middle meatus, and 4 = large polyps causing almost complete nasal obstruction).

### 2.3. Assessment of the Etiology of Olfactory Dysfunction

The etiology of olfactory dysfunction was determined according to clinical history and the results of blood tests, nasal endoscopy, plain radiography, computed tomography, and magnetic resonance imaging [15,16,17,18]. ECRS was diagnosed when the Japanese Epidemiological Survey of Refractory Eosinophilic Chronic Rhinosinusitis (JESREC) score was ≥11. The JESREC score functions as a clinical diagnostic criterion for ECRS. The JESREC score is delineated by four determinants: bilateral lesions (3 points), nasal polyps (2 points), ethmoid-dominant lesions discerned via computed tomography (2 points), and blood eosinophil ratios (%); 0 points for ≤2%, 4 points for 2 < and ≤5%, 8 points for 5 < and ≤10%, and 10 points for 10%< [6]. In addition, a mucosal eosinophil count of ≥70/high-power field (HPF) was histologically confirmed. NCRS was diagnosed when patients had sinusitis but were not diagnosed with ECRS based on the JESREC score. 

### 2.4. Statistical Analysis

Between-group comparisons were performed using the chi-square test, Fisher’s exact test, or the Mann–Whitney U test with Bonferroni correction, as applicable. Quantitative data are presented as mean ± standard deviation (SD). The patients were classified into ECRS, NCRS, and no-CRS groups for comparison. Multivariate logistic regression analyses were performed to investigate factors associated with ECRS, independent of age, sex, and lung function. We compared the etiology of olfactory dysfunction according to FeNO levels. Statistical significance was set at *p* < 0.05. Finally, the prevalence of ECRS was compared according to olfactory dysfunction and FeNO levels. Statistical analyses were performed using JMP Pro version 16 software (SAS Institute Inc., Cary, NC, USA).

## 3. Results

### 3.1. Characteristics of the Participants

A total of 47 patients with asthma were included in this study, 30 of whom had olfactory dysfunction. The patients were divided into three groups: ECRS (*n* = 17), NCRS (*n* = 13), and no-CRS (*n* = 17) (Table 2). The age, sex, BMI, ACT score, ACQ score, forced expiratory volume in 1 s, peak expiratory flow rate, IgE, and presence of allergic rhinitis were not significantly different among the three groups. The FeNO levels (74.22 vs. 28.7 ppb) and blood eosinophil counts (8.5 vs. 4.6%) were significantly higher in the ECRS group than those in the no-CRS group. Patients with ECRS had significantly higher nasal polyp scores (1.9 vs. 0.2 for the right nose and 1.9 vs. 0.1 for the left nose) and prevalence of olfactory dysfunction than those in the no-CRS group (94.1% vs. 35.3%).

### 3.2. Etiology of Olfactory Dysfunction in Asthma Patients with High/Low FeNO Levels

**Figure 1:**
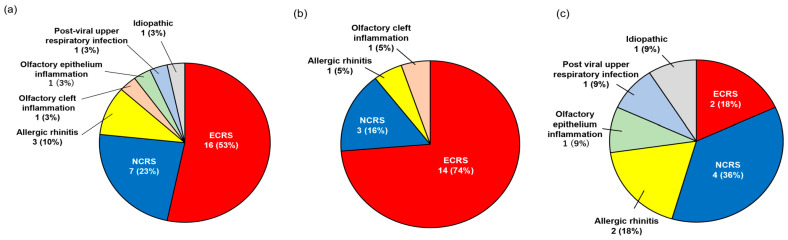
Etiologies of olfactory dysfunction. (**a**) All patients with asthma who had olfactory dysfunction (*n* = 30), (**b**) FeNO ≥ 25 ppb (*n* = 19) and (**c**) FeNO < 25 ppb (*n* = 11). ECRS, eosinophilic chronic rhinosinusitis; FeNO, fractional exhaled nitric oxide; NCRS, non-eosinophilic chronic rhinosinusitis.

CRS was identified as the most prevalent etiology of olfactory dysfunction and was present in 77% (23/30) of the patients with asthma (Figure 1a). Other etiologies were allergic rhinitis (10%), post-viral infection (3%), olfactory cleft inflammation (3%), and olfactory epithelium inflammation (3%). The etiology of olfactory dysfunction for one patient could not be diagnosed; therefore, the case was classified as idiopathic. Univariate and multivariate logistic regression analyses showed that FeNO levels and olfactory dysfunction were risk factors for ECRS (Table 3). In the group with high FeNO levels (FeNO ≥ 25), ECRS (74%, 14/19) was the most common etiology of olfactory dysfunction (Figure 1b); the other etiologies were NCRS (16%), olfactory cleft inflammation (5%), and allergic rhinitis (5%). On the other hand, NCRS (36%, 4/11) was the most common etiology in the low-FeNO-level group (Figure 1c), while other etiologies were ECRS (18%), allergic rhinitis (18%), olfactory epithelium inflammation (9%), post-viral infection (9%), and idiopathic (9%).

### 3.3. Combination of Elevated FeNO Levels and Olfactory Dysfunction Associated with High Risk of ECRS

**Figure 2:**
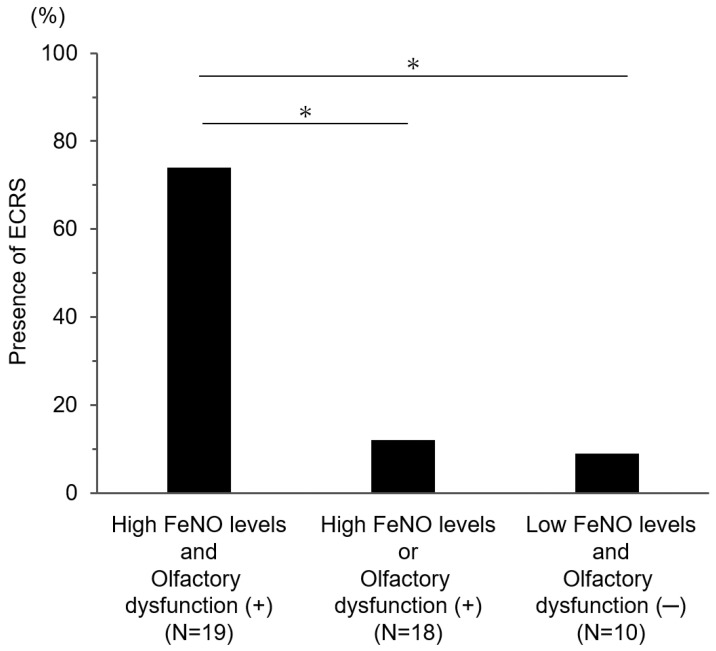
Association of prevalence of ECRS with olfactory dysfunction and FeNO levels (*n* = 47). * *p* < 0.0167; ECRS, eosinophilic chronic rhinosinusitis; FeNO, fractional exhaled nitric oxide.

We compared the prevalence of ECRS among the groups classified according to the presence of olfactory dysfunction and FeNO levels. The prevalence of ECRS was significantly higher in asthma patients with olfactory dysfunction and high FeNO levels (74%) compared with those with either high FeNO levels or olfactory dysfunction and those with low FeNO levels (<25 ppb) and no olfactory dysfunction (12% and 9%, respectively) (Figure 2). The positive predictive value of elevated FeNO and olfactory dysfunction for ECRS was 73%.

### 3.4. Investigation of Olfactory Dysfunction Severity

A total of 80% of olfactory dysfunction cases were evaluated with T&T olfactometry. Olfactory dysfunction is pathologically divided into three categories: i.e., conductive dysfunction, sensorineural dysfunction, and central dysfunction [14]. In the ECRS group, the most severe mean cognitive threshold of 5.8 was observed in 7 out of 14 cases. All of these cases exhibited nasal polyps in both nostrils, which were relatively small in some cases, indicating a potential mix of olfactory dysfunctions other than conductive dysfunction. Conversely, the lowest mean cognitive threshold in the ECRS group was 1.8, encompassing the mild cases. In the ECRS group included seven cases of anosmia and one, three, and three cases of severe, moderate, and mild dysfunction, respectively. The mean and standard deviation of the olfactory cognitive threshold was 4.44 ± 1.60. In the NCRS group, the highest mean cognitive threshold was also 5.8. However, this case was complicated by olfactory epithelium inflammation in conjunction with chronic sinusitis, suggesting a coexisting neurogenic olfactory dysfunction. The NCRS group’s lowest mean cognitive threshold was 1.6. This group included one case of anosmia, two cases of moderate dysfunction, and two cases of mild dysfunction. The mean and standard deviation of the olfactory cognitive threshold was 3.28 ± 1.65. In the no-CRS group, five patients underwent T&T olfactometry assessments. All of them had mild dysfunction. The highest mean cognitive threshold was 2.2, which may have resulted from olfactory cleft inflammation. In contrast, the lowest was 1.4, suggesting allergic rhinitis as the cause. The mean and standard deviation of the olfactory cognitive threshold was 1.80 ± 0.31.

**Figure 3:**
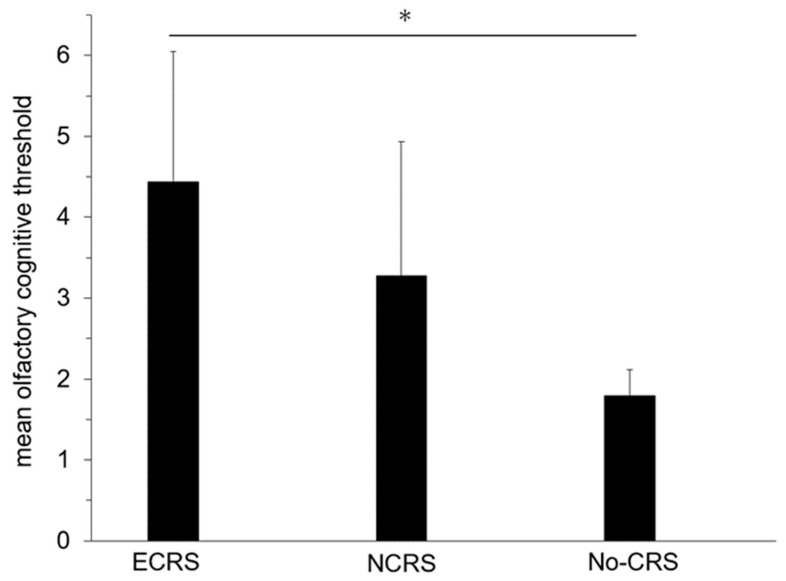
Comparison of mean olfactory cognitive threshold (*n* = 24). * *p* < 0.0167; ECRS, eosinophilic chronic rhinosinusitis; NCRS, non-eosinophilic chronic rhinosinusitis; CRS, chronic rhinosinusitis. Error bars: mean values and SD.

The mean olfactory cognitive threshold, as determined with T&T olfactometry, was notably elevated in the ECRS group compared with the no-CRS group (*p* < 0.025) (Figure 3). Conversely, no substantial differential was observed between the NCRS and no-CRS groups. While the extant literature has posited a heightened severity of olfactory dysfunction in ECRS [18], the present investigation merely discerned a tendency towards an augmented olfactory cognitive threshold in the ECRS group relative to the NCRS group.

## 4. Discussion

This study showed that CRS was the most common etiology of olfactory dysfunction in patients with asthma. Moreover, ECRS was the most common etiology in patients with high FeNO levels, whereas NCRS was the most common etiology in patients with low FeNO levels. Additionally, having both high FeNO levels and olfactory dysfunction were associated with a significantly higher prevalence of ECRS compared with either one of them. The present results showed that both ECRS and NCRS are common etiologies of olfactory dysfunction in patients with asthma and support the link between upper and lower airway inflammation in patients with asthma complicated with olfactory dysfunction. The diagnostic value of NO measurements in patients with BA as a comorbidity has been investigated in a series of studies based on the “one airway/one disease” theory. For example, the use of FeNO in determining the likelihood of steroid responsiveness was strongly recommended for individuals with BA [17].

To the best of our knowledge, this was the first study to report on the etiology of olfactory dysfunction in detail in patients with asthma. In this study, CRS accounted for 77% of the etiologies of olfactory dysfunction, ECRS (53%), and NCRS (23%), with other etiologies including allergic rhinitis, post-viral infection, olfactory cleft inflammation, olfactory epithelium inflammation, and idiopathic. A previous study in general practice reported that the main etiologic diagnoses for olfactory dysfunction were post-viral (34.8%), sinonasal (18.0%), traumatic (15.7%), idiopathic (24.7%), and other (6.7%) in 299 patients with olfactory dysfunction [19]. Another study described the etiologies of olfactory dysfunction as idiopathic (31.5%), rhinitis (28.9%), CRS with polyps (10.5%), and CRS without polyps (7.8%) [2]. A previous study reported more symptom burdens with olfactory dysfunction in patients with asthma complicated with ECRS compared with those of patients without ECRS [20]. The present results demonstrated that NCRS was also a common etiology of olfactory dysfunction in patients with asthma. The extant literature has posited a heightened severity of olfactory dysfunction in ECRS [21]. In the present study, the ECRS group showed a significantly greater severity of olfactory dysfunction than the No-CRS group. This result was consistent with previous reports. Although this study did not find significant differences, the NCRS group also had cases of moderate anosmia, indicating a trend toward greater severity compared with the no-CRS group. 

Olfactory dysfunction is etiologically divided into three categories; conductive dysfunction (i.e., airborne dysfunction caused by sinusitis and nasal allergies), sensorineural dysfunction (i.e., degeneration of the olfactory epithelium and nerves caused by viral infection), and central dysfunction (i.e., disorder of the central nervous system caused by head injury, neurodegenerative diseases, or congenital anomalies) [14]. Olfactory dysfunction is one of the common symptoms of CRS, particularly ECRS. However, the therapeutic outcomes and prognostic factors for the restoration of olfaction after medical treatment or surgical intervention with an endoscopic sinus surgery for ECRS patients remain unclear [6,21]. In this sense, screening procedures to identify underlying CRS morbidity in asthma patients at the earlier stages are of great value and can predict better improvements to retrieve olfactory sensation.

The establishment of classifying CRS endotypes has been attempted, involving histological features such as eosinophilia and specific molecular biomarkers. The integrated analysis of the CRS phenotype and endotype could provide insights into treatment responses and the pathobiology. In particular, classification based on the level of airway NO production and related enzymes has drawn attention with continuing efforts. In this sense, the measurement of FeNO levels and accompanied enzyme activities could provide a foundation for new and specific interventions targeting molecular pathways that underlie endotype-specific inflammation in CRS [22]. 

Nitric oxide synthase (NOS) is an enzyme responsible for NO production from L-arginine to L-citrulline by the action of the NADPH and tetrahydrobiopterin-dependent oxidation [23]. In humans, three NOS isoforms exist: the neuronal (nNOS, NOS1), endothelial (eNOS, NOS3), and inducible (iNOS, NOS2) isoforms. In contrast with nNOS and eNOS, the expression of iNOS in human airways is rather dependent on the activation of various pro-inflammatory cytokines [24]. IL-4 and IL-13 stimulate the synthesis of iNOS in airway epithelial cells via the STAT-6 (signal transducer and activator of transcription-6) pathway, resulting in an augmented production of nitric oxide (NO) [25]. Consequently, patients with asthma, particularly those exhibiting type 2 airway inflammation, present elevated FeNO levels. A significant correlation has been identified between the concentration of exhaled NO in asthma patients and the extent of eosinophilic inflammation in the airways [26]. While the administration of inhaled corticosteroids is known to reduce FeNO levels in patients with asthma, the persistence of elevated FeNO levels, despite corticosteroid inhalation, may indicate corticosteroid-resistant type 2 airway inflammation [27,28,29]. The majority of participants in our study were on inhaled corticosteroids; among those with consistently high FeNO values, ECRS was frequently observed as the predominant factor underlying olfactory dysfunction. 

It is noteworthy that the presence of ECRS has previously been associated with persistent asthma and pronounced eosinophilic inflammation in the lower airways [11,30]. Our previous study on FeNO levels in Japanese patients with ECRS and NCRS demonstrated functional differences in the underlying mechanism of NO production and metabolism [11]. Higher levels of oral and nasal FeNO detected in ECRS patients reflected the persistence of eosinophilic inflammation in paranasal sinus mucosa with concomitant iNOS upregulation and the accompanying deposition of oxidized NO metabolites of nitrotyrosine. Kabayashi et al. recently reported reduced responses to corticosteroids in nasal epithelial cells from ECRS patients with asthma [30]. The responses were associated with decreased phosphatase 2A (PP2A) mRNA expression, a key factor regulating glucocorticoid receptor (GR) nuclear translocation. They proposed that impaired PP2A in nasal polyp tissues may result in reduced GR nuclear translocation and corticosteroid insensitivity. Taken together, our results suggested an association between ECRS-related upper airway inflammation and type 2 inflammation in the lower airways that was resistant to treatment. The results further highlighted that the investigation of FeNO measurements, which is a gaseous and multifunctional transmitter, constitutes a source of fruitful research regarding the human airway system, including both the paranasal sinuses and lower respiratory epithelia.

Prior studies have indicated that increased FeNO levels correlated with elevated eosinophil counts and a decrease in FEV1 among asthma patients [31,32]. Similarly, our study found a positive correlation between FeNO and eosinophil levels and a negative correlation between FeNO and %FEV1 (data not shown). It is known that both asthma and CRS exhibit varied airway inflammatory pathologies [33,34]. In our research, patients with asthma complicated by ECRS had increased FeNO levels, but there was no notable difference in %FEV1 between asthma patients with ECRS and those without CRS. This aligned with a previous report stating no significant FEV1 variation between severe asthma patients with or without accompanying CRSwNP [35]. Our findings suggested that asthma complicated by ECRS correlated with intensified type 2 inflammation in the lower airways. However, the presence of ECRS had a minimal impact on physiological impairments in these airways.

In this study, the prevalence of olfactory dysfunction was high in both ECRS and NCRS (94.1% and 61.5%), which was in agreement with the findings of previous studies that primarily included populations without asthma [36]. In general, olfactory transduction begins when odorants bind to olfactory protein receptors in the olfactory mucosa to generate electrical signals, which are transmitted via the olfactory nerve to the olfactory bulb and finally to the olfactory and orbitofrontal cortexes [37]. Olfactory dysfunction in CRS may be caused by nasal obstruction, which prevents the entry of odorants to the olfactory cleft, or by inflammation of the olfactory epithelium [15,38]. In the present study, NCRS was the most prevalent cause of olfactory dysfunction in asthma patients with low FeNO levels (<25 ppb), indicating a link between T2-low inflammation patterns in the lower and upper airways. Airway inflammation in patients with asthma is heterogeneous, and approximately 40% of patients have a T2-low inflammation pattern [39]. On the other hand, the most prevalent etiology of olfactory dysfunction was ECRS in the high-FeNO group. Moreover, the present results suggested that a combined assessment of FeNO levels and olfactory dysfunction could be used to detect patients with asthma who are at high risk for ECRS [40]. These results supported the link between upper and lower airway inflammatory phenotypes in patients with asthma complicated with olfactory dysfunction. 

In this research, allergic rhinitis was identified in 72.3% of the participants. The incidence of allergic rhinitis has surged markedly since the 1990s [41,42,43] and affects approximately 40% of the global adult population [44]. The concurrent manifestation of allergic rhinitis in bronchial asthma has been posited to exacerbate the ailment’s therapeutic challenges. It is known that asthma is frequently complicated by allergic rhinitis [45]. One plausible rationale for the predominant presence of allergic rhinitis in this study might have been the inclusion criteria, which, in addition to asthma, encompassed participants who visited an otolaryngologist. While the interplay between asthma and allergic rhinitis is acknowledged to influence FeNO [46], the prevalence of allergic rhinitis remained elevated across all groups, with no significant variations discerned.

The limitations of this study included its retrospective design and small sample size. The sample size was relatively small because the patients were selected from those who had asthma, had visited an otorhinolaryngologist, and had undergone some imaging studies. Olfactory dysfunction was evaluated using various methodologies; 16% were evaluated with a medical interview. Self-reported olfactory assessment has been reported to be less sensitive and possibly underestimated in previous studies [47]. Though interviews have been deemed unreliable for certain assessments, numerous publications, primarily those centering on COVID-19-induced olfactory deficits, have relied solely on interview-based evaluations [48,49,50,51,52,53]. Furthermore, certain studies have asserted the reliability of interview-only assessments, particularly concerning profound olfactory dysfunction and olfactory deafferentation [54,55]. In addition, the patients received various treatments, including biologics, making it difficult to determine the influence of each treatment. Finally, this study was conducted at a single institution, and the sample size was relatively small; hence, the results should be validated in a larger cohort. 

## 5. Conclusions

This study showed that both ECRS and NCRS were common etiologies of olfactory dysfunction in patients with asthma. ECRS was the most common etiology of olfactory dysfunction in patients with high FeNO levels, whereas NCRS was the most common etiology in patients with low FeNO levels. The results of this study also suggested that the combined assessment of FeNO levels and olfactory dysfunction could be used to detect patients with asthma who are at high risk for ECRS. These results supported the link between upper and lower airway inflammation in patients with asthma complicated with olfactory dysfunction.

## Figures and Tables

**Table 1:** Diagnostic methods for olfactory dysfunction (N = 30).

T&T olfactometer	24
SNOT-22	1
Only self-reported symptoms	5

**Table 2:** Characteristics of the patients.

	Total	ECRS	NCRS	No-CRS	ECRS vs. No-CRS	NCRS vs. No-CRS
					*p*-Value	*p*-Value
N	47	17	13	17		
Age (yr)	58.0 ± 14.5	57.8 ± 13.2	58.7 ± 15.0	57.6 ± 16.2	0.76	1.00
Female, *n* (%)	26 (55.3%)	8 (47.1%)	8 (61.5%)	10 (58.8%)	0.51	0.90
BMI (kg/m^2^)	24.0 ± 3.6	23.0 ± 3.3	25.8 ± 3.6	23.7 ± 3.6	0.89	0.10
ACT	19.8 ± 5.1	21.3 ± 4.0	19.5 ± 5.5	18.5 ± 5.8	0.12	0.58
ACQ	1.1 ± 1.0	0.8 ± 0.7	1.2 ± 1.3	1.3 ± 1.1	0.30	0.91
FVC (L)	3.1 ± 1.0	3.3 ± 1.1	3.0 ± 0.8	3.0 ± 1.0	0.54	0.93
FEV_1_ (L)	2.1 ± 0.8	2.3 ± 0.9	2.0 ± 0.7	2.1 ± 0.9	0.63	0.90
FEV_1_, % predicted	83.6 ± 21.2	87.1 ± 20.6	81.4 ± 17.8	83.3 ± 24.9	0.84	0.52
FEV_1_/FVC (%)	69.2 ± 11.7	69.0 ± 9.0	68.5 ± 12.2	70.0 ± 14.2	0.51	0.56
FeNO (ppb)	46.9 ± 51.2	74.2 ± 72.7	35.0 ± 24.8	28.7 ± 23.0	<0.025 *	0.29
Eosinophils (/μL)	439.5 ± 557.3	613.8 ± 439.3	335.8 ± 533.5	344.6 ± 660.3	<0.025 *	0.83
Eosinophils (%)	5.9 ± 5.9	8.5 ± 4.7	4.3 ± 5.2	4.6 ± 6.9	<0.025 *	0.72
IgE ^a^ (IU/mL)	345.5 ± 342.2	390.8 ± 444.0	301.3 ± 216.4	322.7 ± 316.4	0.87	0.96
LTRA, *n* (%)	32 (68.1%)	13 (76.5%)	8 (61.5%)	11 (64.7%)	0.47	0.88
LABA, *n* (%)	36 (76.6%)	14 (82.4%)	9 (69.2%)	13 (76.5%)	0.69	0.68
LAMA, *n* (%)	13 (27.7%)	3 (17.6%)	5 (38.5%)	5 (29.4%)	0.44	0.63
Antihistamine, *n* (%)	13 (27.7%)	6 (35.3%)	2 (15.4%)	5 (29.4%)	0.73	0.39
Biologics, *n* (%)	9 (19.1%)	2 (11.8%)	4 (30.8%)	3 (17.6%)	0.65	0.42
Anti-IgE, *n* (%)	3 (6.4%)	0 (0.00%)	2 (15.4%)	1 (5.9%)	0.35	0.88
Anti-IL-5/-5R antibodies, *n* (%)	6 (12.8%)	2 (11.8%)	2 (15.4%)	2 (11.8%)	0.58	0.42
Inhaled corticosteroid, *n* (%)	46 (97.9%)	17 (100.00%)	13 (100.0%)	16 (94.1%)	0.35	0.42
Oral corticosteroid, *n* (%)	10 (21.3%)	2 (11.8%)	3 (23.1%)	5 (29.4%)	0.22	0.72
Pack year	7.2 ± 13.9	5.4 ± 12.5	7.3 ± 15.6	9.0 ± 14.5	0.65	0.98
Nasal polyp score (right)	0.8 ± 1.1	1.9 ± 1.0	0.2 ± 0.6	0.1 ± 0.3	<0.025 *	0.75
Nasal polyp score (left)	0.7 ± 1.1	1.9 ± 1.1	0.1 ± 0.3	0.1 ± 0.3	<0.025 *	0.75
Atopic factor, *n* (%) ^b^	28 (59.6%)	13 (76.5%)	4 (30.8%)	11 (64.7%)	0.17	0.42
Allergic rhinitis, *n* (%)	34 (72.3%)	13 (76.5%)	7 (53.8%)	14 (82.4%)	0.69	0.10
Nasal discharge, *n* (%)	23 (48.9%)	11 (64.7%)	7 (53.8%)	5 (29.4%)	0.04	0.19
Nasal obstruction, *n* (%)	21 (44.7%)	10 (58.8%)	5 (38.5%)	6 (35.3%)	0.18	0.88
Olfactory dysfunction, n (%)	30 (63.8%)	16 (94.1%)	8 (61.5%)	6 (35.3%)	<0.025 *	0.17

The data are presented as the mean ± SD unless otherwise stated. ^a^ *n* = 41. ^b^ *n* = 32. * *p* < 0.025, Mann-Whitney U-test with Bonferroni correction. Abbreviations: ACT, asthma control test; ACQ, asthma control questionnaire; BMI, body mass index; CRS, chronic rhinosinusitis; ECRS, eosinophilic chronic rhinosinusitis; FeNO, fraction exhaled nitric oxide; FVC, forced vital capacity; FEV_1_, forced expiratory volume in 1 s; LABA, long-acting beta 2 agonist; LAMA, long-acting muscarinic antagonists; LTRA, leukotriene receptor antagonist; NCRS, non-eosinophilic chronic rhinosinusitis; SD, standard deviation.

**Table 3:** Univariate and multivariate logistic regression analyses to identify factors associated with having ECRS in all participants (*n* = 47).

	Univariate Analysis			Multivariate Analysis		
	OR	95% CI	*p*-Value	OR	95% CI	*p*-Value
Age	0.99	0.95–1.04	0.93			
Female	0.59	0.17–1.96	0.39			
BMI	0.87	0.71–1.04	0.12			
ACT	1.10	0.97–1.28	0.12			
ACQ	0.59	0.27–1.13	0.12			
FEV1, % predicted	1.00	0.97–1.03	0.61			
FEV1/FVC	0.99	0.94–1.05	0.91			
FeNO	1.02	1.00–1.05	<0.05	1.03	1.01–1.07	<0.05
FeNO ≥ 25 ppb (versus < 25 ppb)	8.06	1.88–34.40	<0.05	13.27	1.80–97.68	<0.05
Eosinophils (/μL)	1.00	0.99–1.00	0.11			
Allergic rhinitis	1.39	0.36–5.99	0.63			
Olfactory dysfunction	18.28	3.11–351.35	<0.05	15.21	2.04–329.44	<0.05
Nasal discharge	2.75	0.82–9.95	0.10			
Nasal obstruction	2.47	0.74–8.66	0.14			

ACT, asthma control test; ACQ, asthma control questionnaire; BMI, body mass index; ECRS, eosinophilic chronic rhinosinusitis; FeNO, fractional exhaled nitric oxide; FEV_1_, forced expiratory volume in 1 s.

## Data Availability

The data that support the findings of this study are available from the corresponding author upon reasonable request.
